# An Extremely Preterm Infant With *PIK3CA-*Related Overgrowth Spectrum (PROS): Alpelisib Treatment and Outcome

**DOI:** 10.1155/crig/6839348

**Published:** 2025-09-30

**Authors:** Sharon Anderson, Milen Velinov

**Affiliations:** ^1^Medical Genetics, Rutgers Health, Rutgers Robert Wood Johnson Medical School, New Brunswick, New Jersey, USA; ^2^Division of Advanced Nursing Practice, Rutgers School of Nursing, Newark, New Jersey, USA

**Keywords:** macrocephaly-capillary malformation syndrome, megalencephaly, megalencephaly-capillary malformation-polymicrogyria syndrome, megalencephaly-capillary malformation syndrome, PIK3CA-related overgrowth spectrum

## Abstract

Phosphatidylinositol 4,5-bisphosphate 3-kinase catalytic subunit alpha (*PIK3CA*)-related overgrowth spectrum (PROS) is a group of rare genetic asymmetric and atypical overgrowth disorder syndromes. Affecting skin, adipose and connective tissues, brain, bone, and vasculature and severity influenced by the gestational age at which the change occurred, PROS is phenotypically heterogeneous. This paper shares the case report of a former extremely preterm infant diagnosed with a subtype of PROS, megalencephaly-capillary malformation/megalencephaly-capillary malformation polymicrogyria (MCAP) syndrome, for whom treatment with alpelisib was initiated at 10 months of age (7 months corrected age). To our knowledge, this patient is the third and youngest to be included in this expanded access program for compassionate use for patients under 2 years of age.

## 1. Introduction

Phosphatidylinositol 4,5-bisphosphate 3-kinase catalytic subunit alpha (*PIK3CA*)-related overgrowth spectrum (PROS) is a group of rare genetic asymmetric and atypical overgrowth disorder syndromes caused by variants in the *PIK3CA* gene mapped to 3q26.3 (MIM 171834) [1]. Although poorly studied and likely underestimated, the incidence is approximately 1 in 22,000 [2]. Associated subtypes result from postzygotic variants that alter the p110α protein, a subunit of the enzyme phosphatidylinositol 3-kinase (PI3K). Gain of function of *PIK3CA* results in increased cell division and overgrowth in skin, adipose, connective tissue, brain, bone, and vasculature cells [3–6]. PROS is a phenotypically heterogeneous spectrum of disorders [6]. Disease severity is influenced by the gestational age at which the change occurred (the earlier the change, the greater the level of mosaicism), the location and type of the affected tissue, and environmental factors [7, 8]. The growth rate depends on the variant type, degree of mosaicism, and virulence/oncogenicity [9]. Heterogeneity among affected individuals with PROS, even within the same subtype, necessitates management recommendations and guidelines tailored to each patient [10].

Here, we present the case of a former extremely preterm infant diagnosed with a subtype of PROS, megalencephaly-capillary malformation/megalencephaly-capillarymalformation-polymicrogyria (MCAP) syndrome. In addition to supportive care, this patient was offered a targeted PROS therapy, alpelisib [11]. We provide a brief overview of MCAP syndrome, followed by a summary of our patient's past medical and clinical course over the first year of treatment.

### 1.1. Megalencephaly-Capillary Malformation Syndrome

Over the years, this condition has been described by several names, including macrocephaly-cutis marmorata telangiectatica congenita (M-CMTC), macrocephaly-capillary malformation (M-CM), and currently, MCAP syndrome. Characterized by megalencephaly and capillary malformations, at least 15 *PIK3CA* variants are known to cause MCAP [5]. Meganencephaly is typically noted at birth, and the head continues to overgrow during the first few years of life before growth slows [4]. Brain abnormalities are common and may include hydrocephalus, markedly thick corpus callosum, cerebellar tonsillar ectopia with crowding of the posterior fossa (Chiari malformation), and syringohydromyelia. When present, perisylvian polymicrogyria exacerbates feeding/swallowing difficulties and speech delays [3, 5, 12]. The range of intellectual disability (mild to severe) is related to the severity of cortical dysplasia/polymicrogyria, hydrocephalus, and seizures [6]. Seizures affect approximately 30%–40% of individuals with *PIK3CA* pathogenic variants, differing in type and severity depending on the variant distribution and the presence or absence of associated cortical malformations [6]. Vascular malformations such as persistent nevus simplex are typically seen on the face (nose), upper lip, and philtrum [4, 6]. Some appear as webbed patches resembling cutis marmorata [5]. Less commonly, arterial or mixed capillary-lymphatic-venous or arteriovenous malformations may be present [12]. There can also be segmental overgrowth/asymmetry of limbs/extremities and cutaneous syndactyly [4, 8, 12]. Due to connective tissue dysplasia, there can be joint hypermobility and skin hyperelasticity and laxity [6]. Although endocrine problems affect only a small percentage of those affected, individuals may experience hypoinsulinemic hypoketotic hypoglycemia, hypothyroidism, and growth hormone deficiency [5]. While non-MCAP PROS has been associated with Wilms tumor, leukemia, vestibular schwannoma, retinoblastoma, and meningiomas, which suggests possible tumorigenesis at cancer-related hotspots, individuals with cell overgrowth associated with MCAP do not appear to have an increased cancer risk [3, 5, 8, 13].

Because there are several types and severity varies, treatment rests with close surveillance and intervention based on patient-specific symptoms/clinical findings. Notably, affected individuals may have only some of the symptoms described [14]. Megalencephaly, hemimegalencephaly, and/or cortical dysplasia, polymicrogyra, Chiari malformation, and ventriculomegaly/obstructive hydrocephalus must be monitored. Treatment/therapies are based on symptomology and associated neurological problems such as hypotonia, seizures, autistic features, mild to severe intellectual disability, and behavioral problems [6]. Subcutaneous lipomas and lymph malformations may require compression garments, lymphatic massage, and/or surgical and debulking procedures. Interventional radiological procedures may be used to restrict blood flow to affected areas. Sclerotherapy and laser therapy may help address cutaneous vascular malformations on the face and cutis marmorata. Individuals with vascular and/or lymphatic anomalies may be treated with sirolimus (rapamycin). While these mTOR inhibitors stop cellular proliferation, a nonrandomized open-label pilot treatment study of 39 individuals (three with MCAP) did not assess outcome measures for low-dose sirolimus on CNS-related malformations and neurologic complications associated with MCAP and noted that the side-effect profile is significant [15]. More promising is a new medication for the systemic treatment of PROS called alpelisib, which has been approved by the U.S. Food and Drug Administration (FDA) following accelerated approval. In addition to supportive care, the following case presentation shares the clinical course and outcome for our patient who received alpelisib treatment.

## 2. Case Presentation

Our genetics team was asked to consult on this patient at 8 months of age (approximately 5 months corrected age) when she was admitted from a neighboring pediatric rehabilitation facility because of bilateral ventriculomegaly, megalencephaly with a full anterior fontanelle and separated sutures, and seizures related to ventricular peritoneal (VP) shunt malfunction. Post grade IV intraventricular hemorrhage (IVH), she was a former 26 + 6/7 week gestational age infant with a history of respiratory distress syndrome (now chronic lung disease), facial dysmorphism (long face with parietal bossing, facial asymmetry, slight hypotelorism, bilaterally low-set ears), congenital anomalies (2 vessel cord, agenesis of the corpus callosum), nystagmus, cardiomegaly, history of central venous thrombus, hemangioma (back), and PROS. Other problems included milk protein intolerance (treated with Nutramigen Probiotic LGG formula, Mead Johnson & Company, LLC, Chicago, IL) and dependence on a gastrostomy tube. The infant was receiving 1–2 L of oxygen via a nasal cannula. Medications included albuterol, budesonide, levetiracetam, and famotidine. Diazepam and midazolam were available if needed. An overview of the MCAP phenotype and features identified for our patient based on her history at our baseline visit is provided in Table 1.

### 2.1. Relevant Past Medical and Neonatal Intensive Care Unit History

Performed during her neonatal intensive care unit stay, the initial MRI showed severe hydrocephalus with dilation of the lateral and third ventricles consistent with communicating hydrocephalus. There was evidence of an IVH with casts of blood in the lateral ventricles and a small volume of dependent blood in the left atrium/occipital horn. Postcontrast imaging showed enhancement along the ependymal surfaces of the lateral ventricles, which was consistent with IVH. Agenesis of the corpus callosum and limited sulcation of the brain with a thickened cortical mantle, suspicious of migration disorder or lissencephaly, were noted.

An overgrowth and macrocephaly syndromes gene panel identified mosaicism for a pathogenic variant in the *PIK3CA* gene (c.1093 G > A, p.Glu365Lys). Of note, a variant of uncertain significance in the *TFBN3* gene (c.730 G > A, p.Glu244Lys) associated with autosomal dominant arrhythmogenic right ventricular dysplasia and Loeys–Dietz syndrome was also identified. Microarray showed heterozygosity for an ∼379 kb duplication at 4p16.1 (a variant of uncertain significance), and Beckwith–Weidemann/Russell–Silver syndrome methylation studies were normal.

### 2.2. Relevant Postneonatal Intensive Care History

There were several admissions to the birth hospital for ventriculomegaly and VP shunt malfunction requiring external ventricular drain (EVD) placement and shunt revision. Given that the hospitalizations were outside our healthcare system, finding and following the prehospitalization course, diagnosis, and imaging was challenging. To the best of our ability, we have identified and extrapolated the relevant components.

The day before this admission, she was seen in the emergency department for seizures, and the levetiracetam dose was increased. On the day of admission to our adjacent hospital, there was cerebrospinal fluid leakage from the previous EVD site. The left ventricular catheter and right VP shunt were removed, and again, an EVD was placed. Ultimately, the VP shunt was replaced. It was during this hospitalization that our genetics team was consulted.

### 2.3. Treatment Recommendations

During our inpatient consultation, we discussed alpelisib, a newly FDA-approved medication for individuals with *PIC3CA* variants over 2 years of age. We discussed the safety and effectiveness of alpelisib in children under 2 years has not been established, and there are no data regarding neurological treatment efficacy. After weighing the treatment risks and benefits, the parents decided to move forward with treatment. We pursued insurance coverage for compassionate use (Novartis' Managed Access Programs, Novartis Pharmaceuticals Corporation), and alpelisib was initiated after discharge at 10 months of age (7 months corrected age). Given the patient's age, we prescribed 25 mg (1/2 of a 50 mg tablet) daily.

### 2.4. Alpelisib

Alpelisib is an FDA-approved kinase inhibitor that works upstream to sirolimus [16]. With a primary outcome measure that included at least a 20% reduction in target lesions from baseline, and no lesion with greater than or equal to a 20% increase from baseline, the *EPIK-P1* study showed alpelisib-reduced PROS lesion size and demonstrated clinically meaningful and durable reduction in the size of PROS lesions in patients with severe disease who required systemic therapy [17–19]. Based on this evidence, accelerated approval for adult and pediatric patients with severe PROS who require systemic therapy over 2 years of age was granted. Current evidence suggests alpelisib slows the growth of existing lesions and prevents new lesions [11, 16]. Because this medication is part of a chemotherapy regimen for hormone receptor (HR) positive and human epidermal growth factor receptor 2 (HER2) negative *PIK3CA*-mutated advanced or metastatic breast cancer in combination with fulvestrant for postmenopausal women and male patients, the pharmacodynamics and pharmacokinetics have not yet been studied in pediatric patients [16]. Furthermore, while alpelisib is FDA-approved to reduce overgrowth and vascular lesions, the reduction of functional complications has yet to be demonstrated, and the efficacy of treating neurologic findings associated with MCAP syndrome remains unknown [6]. At the writing of this manuscript, FDA approval was based on a single-arm clinical study of pediatric and adult patients with PROS under an expanded access compassionate use program. A larger phase 2, placebo-controlled prospective study is ongoing and will provide additional evidence. The multicenter clinical trial incorporates an initial 16-week, randomized, double-blind, placebo-controlled period followed by extension periods to assess the efficacy, safety, and pharmacokinetics of alpelisib in adult and pediatric participants with PROS [11].

Established dosing recommendations exist for adults 18 years and older (250 mg daily) and recommendations for two pediatric patient groups. Specific to younger children and based on the primary analysis for efficacy, children 2–5 years of age receive 50 mg of alpelisib. Individuals aged 6–17 years receive an initial dose of 50 mg. If there is no response after 24 weeks of treatment, the dose may be increased to 125 mg daily. The most common adverse reactions include diarrhea, stomatitis, hyperglycemia, headache, dry skin, and alopecia [11, 16, 19]. Other reactions include hypersensitivity, skin reactions, colitis, pneumonitis, and embryo-fetal toxicity [16]. If adverse reactions are experienced, dosage modification or discontinuation recommendations are available.

### 2.5. Clinical Course and Treatment Response

#### 2.5.1. First Outpatient Visit

The first outpatient visit with our genetics center was at 11 months of age (8 months corrected). Since our initial consultation and hospital discharge, there were three hospitalizations at the birth hospital (bronchiolitis secondary to rhino-/enteroviruses, respiratory syncytial virus, and coronavirus and seizures) and one at another outside hospital (seizures). Several specialist follow-up appointments were missed due to recurrent illnesses/hospitalizations. At the time, she was receiving GT feedings of Similac Alimentum (Abbott) due to supply chain problems, oxygen via nasal cannula, albuterol, budesonide, and levetiracetam with PRN acetaminophen, diazepam, and midazolam. She was receiving 8 hours of home nursing care.

**Figure 1:**
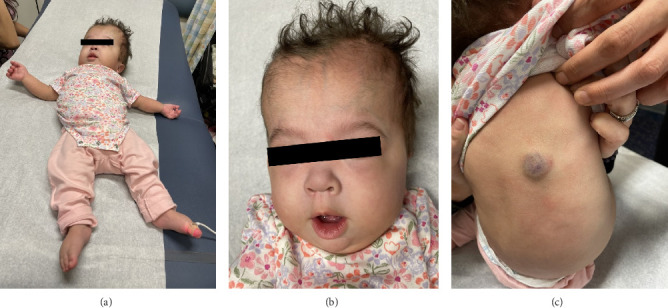
Former extremely preterm infant with megalencephaly-capillary malformation/megalencephaly-capillary malformation polymicrogyria (MCAP) syndrome. *Note:* the above images were taken at 11 months (8 months corrected) age during her first outpatient visit with our genetics center. Note the generalized hypotonia (a); megalencephaly, frontal bossing (b); and involuting hemangioma on the lower left scapula (c).

No adverse events to alpelisib were noted after 3 months of treatment. On examination (see Figure 1), she had significant developmental delays, including severe hypotonia and minimal spontaneous movements. She was unable to hold a pacifier. The hemangioma on her scapula was starting to involute. The extremity size was reasonably symmetrical. Her growth parameters (corrected for prematurity) were as follows: weight 8.74 kg (75.08% tile), length 70 cm (62.66% tile), ad head circumference 49.5 cm (> 99% tile, ∼90% tile for a 20-month-old).

We discussed the overlap of the patient's developmental and neurological problems related to prematurity/IVH, megalencephaly associated with *PIK3CA*, and agenesis of the corpus callosum and that it is unclear if the IVH was a result of prematurity, a vascular abnormality associated with PROS, or a combination of both. Developmental challenges are most likely related to a combination of these overlapping contributing factors.

We discussed our treatment goals to minimize/prevent overgrowth with alpelisib therapy while preventing seizures and maintaining intraventricular pressure low to optimize development. Due to the mosaic nature of this condition, we discussed the importance of ongoing follow-up and surveillance to assess her therapeutic response to alpelisib. In addition to neurology and neurosurgery, we recommended follow-up consultation with several specialists, including gastroenterology, a dietitian, pulmonology, cardiology, ophthalmology, and endocrinology. Other recommendations included blood work (IGF1, prolactin, CBC, and CMP) and an MRI to monitor her treatment response before follow-up in 6 months.

#### 2.5.2. Second Outpatient Visit

During the second outpatient visit with our genetic center, the patient was 19 months (16 months corrected age). There had been several emergency department visits for GT replacements and hospitalizations at the birth hospital, including a prolonged admission (2 1/2 months). Palliative care was reintroduced, and when ready for discharge, she was transferred to a pediatric long-term care facility where she currently resides. Several follow-up appointments with our center were missed because of recurrent illnesses/hospitalizations.

An overview of the interim history and current health status is as follows. Her primary neurological and neurosurgical problems related to seizures and shunt malfunction were improved. Multiple imaging studies were performed 4 months prior to the visit. Agenesis of the corpus callosum, frontoparietal polymicrogyria, and Chiari malformation were unchanged. CSF density/cystic lesions remained stable, and the cardiac size was normal. There was a concern about pneumonitis, a side effect of alpelisib, which would have required discontinuation. Radiographs, however, eliminated it from the differential. Several tissue and vascular studies were completed, and no abdominal/hepatic/renal masses/lesions or vascular lesions, or venous thrombosis were seen.

On exam, she was nonverbal, but providers reported she smiled and laughed. Additional dysmorphic features included a flattening of the face and severe horizontal nystagmus. Her development remained significantly delayed. Severe hypotonia persisted, and reflexes were absent. She could hold her thumb in her mouth with her left arm but could not lift her right arm. She remained GT feeding dependent. Medications included the addition of vitamin D3, clonidine, and lorazepam. She had been receiving alpelisib for approximately 1 year. She experienced one episode of hypoglycemia, for which a comprehensive evaluation was completed. Two weeks before this visit, she was seen by our pediatric endocrinologist, and another tier of testing was completed (see Table 2). It is the opinion of the pediatric endocrinologist that the event was most likely nonketotic hyperinsulinemic hypoglycemia with some element of late dumping syndrome after gastrostomy tube feeding. A recent IGF1 was 17 (low), and CBC and CMP were essentially normal. To date, there have been no adverse events, and her neurological and neurosurgical status has stabilized after treatment. Her growth parameters were as follows (percentile for corrected age): weight 11.3 kg (86.97% tile), length 76 cm (16.64% tile), and head circumference 52 cm (> 99% tile, ∼50% tile for a 9-year-old). Notably, there was a discrepancy in the length obtained during the visit, as her length at 18 months of age obtained during her endocrinology appointment was 78 cm (49.59% tile). Table 1 provides an overview of the evolution of the MCAP phenotype and features identified in our patient after 1 year of treatment. Table 2 provides an overview of relevant laboratory testing and results at baseline, after initiation of treatment, and after 7–8 months and 1 year of treatment.

## 3. Conclusion

To our knowledge, this is the third and youngest patient to receive alpelisib through the expanded access program for compassionate use for patients < 2 years of age. While this patient has not demonstrated robust improvement in cognitive development after 1 year of treatment, alpelisib cannot reverse the previous neurological insult associated with megalencephaly, ventriculomegaly, agenesis of the corpus callosum, seizures, extreme prematurity, and IVH. With continued treatment and therapies provided at the inpatient facility within which she resides, her medical status and growth have stabilized, and she is making slow developmental progress with no adverse treatment effects. Based on these findings, the plan is to continue alpelisib, provide ongoing supportive care, and facilitate ongoing surveillance in collaboration with her residential medical team.

## Figures and Tables

**Table 1:** Phenotype comparison of megalencephaly-capillary malformation (MCAP) syndrome and our patient at baseline and after one year of treatment.

MCAP phenotype	Baseline	One year
*Brain/neurological*		
Meganencephaly	+	+
Hydrocephalus	+	+
Thick corpus callosum	Absent	Absent
Chiari malformation	+	+
Syringohydromyelia	NA	−
Perisylvian polymicrogyria	+	+
Hypotonia	+	+
Developmental delay/intellectual disability	+	+
Seizures	+	+
Feeding/swallowing difficulties	+	+
Speech delay	NA	+

*Tissue and vascular*		
Overgrowth/asymmetry of limbs/extremities	−	−
Cutaneous syndactyly	−	−
Vascular malformations	Capillary hemangioma	Involuting
Arterial or mixed capillary-lymphatic-venous or arteriovenous malformations	−	−

*Endocrine*		
Hypoinsulinemic hypoketotic hypoglycemia	+	+/−
Hypothyroidism	+	−
Growth hormone deficiency	+	−

*Skin and joints*		
Skin laxity/hyperelasticity	−	−
Joint hypermobility	+	+

*Note:* + = present, − = absent, +/− = may or may not be present/absent, NA = not applicable/not available/unable to be assessed.

**Table 2:** Relevant laboratory testing in response to alpelisib treatment: baseline to one year of age.

Test (normal range)	Baseline	1–2 weeks of treatment	7–8 months of treatment	1 year of treatment
Cortisol (morning 3.0–25.0 μg/dL)	20.30		23.0	
GH (0.0–10.0 ng/mL)	2.6			0.9 ↓
IGF I (female, normal based on age, ng/mL)		12 ↓, < 10 ↓ (< 1 year, 14–106)		17 ↓ (1–2 years, 23–136)
IGFBP3 (normal based on age, ug/L)	462 ↓ (0–11 months, 1053–3271)	392 ↓ (1 year, 1221–3721)		1164 (1 year, 1221–3721)
Prolactin (2.0–23.0 mg/mL)	25.0 ↑	25.0 ↑, 19.1		
T3 (60–181 ng/dL)		158, 115		175
Free T3 (2.3–4.20 pg/mL)				3.80
Free T4 (0.9–1.8 ng/dL)	1.29	0.928		1.12
TSH (0.35–5.5 UIU/mL)	8.50 ↑	3.16		4.10
AFP marker (0.5–11.1 ng/mL)			8.6	
Insulin (2.6–24.9 µIU/mL)	0.0 ↓			
Beta-hydroxybutyrate (0.2−2.8 mmol/L)	0.70			
CMP	Glucose 53 ↓ (70–100 mg/dL) TP 4.9 ↓ (6–8 g/dL) Alb 3.4 ↓ (3.5–5.5 g/dL)		Unremarkable	
CBC			WBC 4.1 ↓ Hct 32.1 ↓ Otherwise unremarkable	

*Note:* T3 = triiodothyronine, T4 = thyroxine, ↑ = high, ↓ = low.

Abbreviations: AFP = alpha fetoprotein, CBC = complete blood count, CMP = comprehensive metabolic panel, GH = growth hormone,

IGF = insulin-like growth factor, IGFBP3 = insulin-like growth factor binding protein 3, TSH = thyroid stimulating hormone.

## Data Availability

No personal data processing was required for this case report.
